# Novel Avian Influenza A(H7N9) Virus in Tree Sparrow, Shanghai, China, 2013

**DOI:** 10.3201/eid2005.131707

**Published:** 2014-05

**Authors:** Baihui Zhao, Xi Zhang, Wenfei Zhu, Zheng Teng, Xuelian Yu, Ye Gao, Di Wu, Enle Pei, Zhengan Yuan, Lei Yang, Dayan Wang, Yuelong Shu, Fan Wu

**Affiliations:** Shanghai Municipal Center for Disease Control and Prevention, Shanghai, China (B. Zhao, X. Zhang, Z. Teng, X. Yu, Y. Gao, Z. Yuan, F. Wu);; National Institute for Viral Disease Control and Prevention, Chinese Center for Disease Control and Prevention, Beijing, China (W. Zhu, L. Yang, D. Wang, Y. Shu);; Shanghai Wildlife Conservation and Management Center, Shanghai (D. Wu, E. Pei)

**Keywords:** H7N9, tree sparrow, wild bird, influenza, viruses, China

## Abstract

In spring 2013, influenza A(H7N9) virus was isolated from an apparently healthy tree sparrow in Chongming Dongping National Forest Park, Shanghai City, China. The entire gene constellation of the virus is similar to that of isolates from humans, highlighting the need to monitor influenza A(H7N9) viruses in different species.

Since its emergence in China in February 2013, avian influenza A(H7N9) virus has resulted in 217 human infections and 57 deaths (*1*). The biological features of the virus and its pandemic potential have caused global concern (*2*). Although the epidemic declined quickly after the closure of live poultry markets in China in April 2013, new cases in humans have reemerged since October 2013. The number of new cases has increased sharply since January 1, 2014, paralleling the peak of the first wave (*1*,*3*,*4*), indicating that subtype H7N9 viruses were circulating asymptomatically among natural hosts. Sequence data indicated that the hemagglutinin gene of this novel subtype H7N9 virus might originate from a subtype H7N3 virus in ducks and that the neuraminidase gene probably originated from a subtype H7N9 virus in wild birds (*5*) or ducks or chickens (*6*,*7*). These data suggest that wild birds might play a role in the emergence of subtype H7N9 viruses, similar to the role they played in the geographic spread of avian subtype H5N1 viruses (*8*). However, although avian influenza A(H7N9) viruses have been isolated from chickens and pigeons, to our knowledge, none have been isolated from wild birds. To better understand the role of wild birds in the emergence and potential dissemination of subtype H7N9 viruses, during spring of 2013, the Shanghai Municipal Center for Disease Control and Prevention, in collaboration with the Shanghai Wildlife Conservation and Management Center, investigated influenza A(H7N9) virus infection among wild birds in Shanghai. 

## The Study

**Figure 1:**
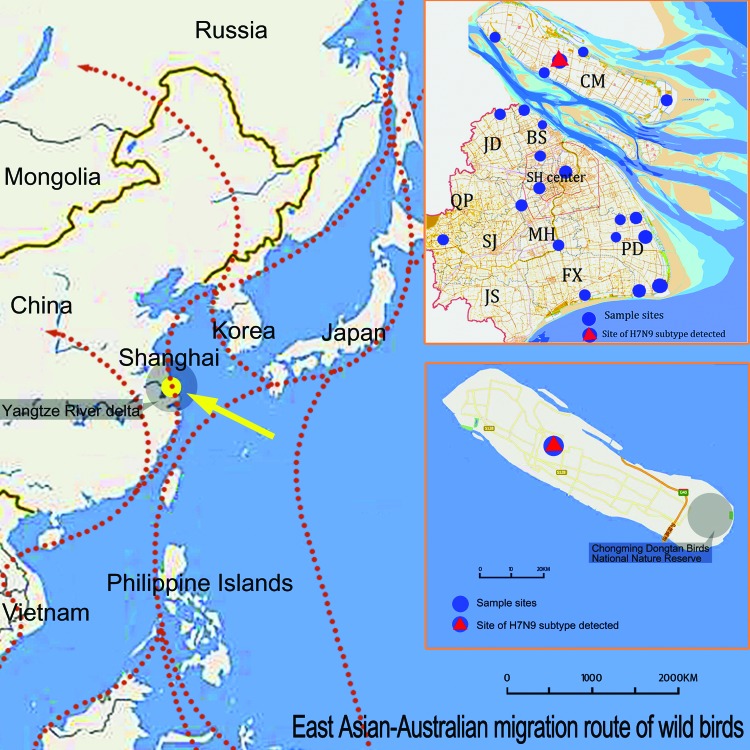
Location of tree sparrow from which novel avian influenza A(H7N9) virus was isolated: Chongming National Dongping Forest Park of Shanghai (yellow solid circle), which is located in the Australia–East Asia migratory wild bird flyway. Top right: sampling locations in Shanghai City. Bottom right: sampling location of influenza A(H7N9)–positive tree sparrow. CM, Chongming district; BS, Baoshan District; JD, Jiading District; SH center, Changning, Putuo and Xuhui Districts; QP, Qingpu District; MH, Minhang District; SJ, Songjiang District; PD, Pudong District; FX, Fengxian District; JS, Jinshan District.

During April 10–May 15, a total of 2,198 fecal, tissue, cloacal swab, and tracheal swab samples were collected from wild birds in Shanghai. Trained staff captured healthy birds with an approved trapping method, collected samples, and released the birds. Tissue samples were collected from naturally dead wild birds. Information on bird species and sampling places are listed in Technical Appendix Tables 1 and 2). RNA was extracted from each sample and tested by using influenza A universal real-time PCR according to the standard operating procedure of the World Health Organization (*9*). Influenza A virus–positive specimens were further subtyped by reverse transcription PCR with an avian influenza A virus subtype primer set reported previously (*10*). Of the 2,198 samples, 28 were positive for influenza A virus. One tracheal sample from an apparently healthy tree sparrow was positive for the novel subtype H7N9 virus, whereas the cloacal swab samples from this bird were negative. The positive sample was inoculated into 11-day-old specific pathogen free embryonated chicken eggs for virus isolation. The isolated virus was termed A/tree sparrow/Shanghai/01/2013 (H7N9). The tree sparrow had been collected from a forest on Chongming Dongping Forest Park, which is 47 km from Dongtan National Nature Reserve, a winter habitat for wild migratory birds (Figure 1).

To explore the genetic relationships between this sparrow-derived influenza A(H7N9) virus and other viruses from humans and poultry, we amplified total genomic segments by using viral RNA directly isolated from the original specimen with the primer sets listed in Technical Appendix Table 3 and sequenced by Sunny Biotech Co., Ltd. (Shanghai, China). The Chinese National Influenza Center performed the sequencing by using RNA from chicken embryonated cultured viruses in an ABI 3730xl automatic DNA analyzer (Life Technologies, Foster City, CA, USA). Full-genome sequences from the original sample and the embryonated chicken eggs isolation were deposited in GenBank under accession nos. KF609524–KF609531 and KJ508887-KJ508894, respectively. To facilitate the phylogenetic analysis, we downloaded sequences of the novel subtype H7N9 viruses from 2013 and the avian subtype H7N9 viruses from before 2013 from the Global Initiative on Sharing Avian Influenza Data (http://platform.gisaid.org/epi3/frontend#46b284). Sequence alignments were performed by using the MegAlign method of Lagergene 7.01 software (www.dnastar.com/t-megalign.aspx). Phylogenetic analysis was analyzed by using the neighbor-joining method in MEGA software version 5.10 (www.megasoftware.net).

**Figure 2:**
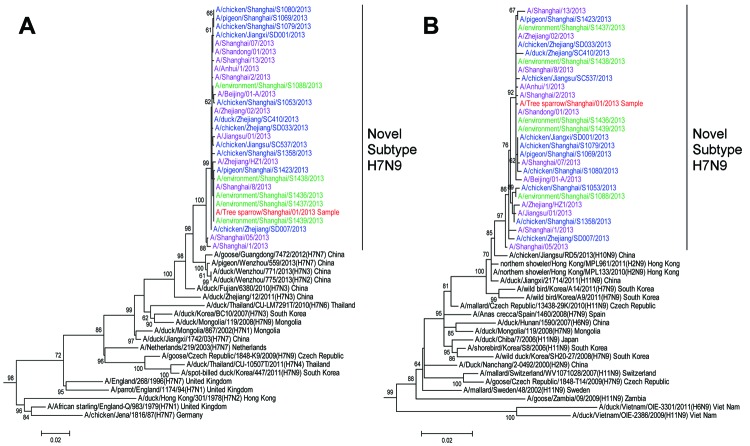
Phylogenetic tree of the hemagglutinin (A) and neuraminidase (B) genes of influenza A(H7N9) viruses. Multiple alignments were constructed by using the MUSCLE algorithm of MEGA software version 5.10 (www.megasoftware.net). Phylogenetic trees were constructed by using the neighbor-joining method with bootstrap analyses of 1,000 replications. Bootstrap values >60% are shown in the nodes. Sequences of human influenza A(H7N9) viruses are shown in purple, novel subtype H7N9 viruses from poultry (chickens, ducks, and pigeons) in blue, novel subtype H7N9 viruses from the environment in green, and novel subtype H7N9 viruses from wild birds in red. Scale bar indicates base substitutions per site.

Eight gene segments of the tree sparrow virus shared most (>99.3%) similarities with subtype H7N9 virus isolates from humans. Phylogenetic analysis of hemagglutinin genes revealed that subtype H7N9 viruses could be classified into Eurasia and North America lineages. The subtype H7N9 virus in this study shared the same influenza lineage with all novel subtype H7N9 viruses from humans and poultry (Figure 2). Like the hemagglutinin genes, the other 7 genes showed the same evolutionary pattern (Technical Appendix Figure 1). Homology and phylogenetic analyses indicated that the genetic constellation of the tree sparrow–derived subtype H7N9 virus is similar to that of novel subtype H7N9 avian influenza viruses isolated from humans and poultry in this region.

According to genetic signatures, the tree sparrow–derived subtype H7N9 virus acquired the ability to bind to human-like receptors, for which substitutions G186V and Q226L in hemagglutinin protein (H3) are responsible, similar to most human and avian subtype H7N9 viruses. A 69-73–aa deletion was also found in its neuraminidase gene (N2). Amino acid 292R was maintained in neuraminidase genes, indicating its sensitivity to neuraminidase inhibitors. However, an S31N mutation in the matrix 2 protein confers resistance to adamantine. Asp at polymerase basic 2 protein (PB2) residue 701 was associated with reduced transmissibility. Mixed E/K at residue 627 in PB2 and V/I at residue 31 in matrix 1 protein were detected from the original sample (Technical Appendix Figure 2). Because all previously reported influenza A (H7N9) viruses isolated from birds or the environment acquired PB2 627E, the mixed amino acids of 627E/K from direct sequencing of the tree sparrow's original tracheal swab sample suggested that the PB2 E627K substitution might have occurred during replication of the virus in birds.

## Conclusions

The high similarity of genes from the avian influenza A(H7N9) virus from an apparently healthy tree sparrow in Shanghai and influenza A(H7N9) viruses from humans and poultry in this region indicate that avian influenza A(H7N9) virus might be transmitted from poultry to tree sparrows or vice versa. Earlier reports documented that influenza A viruses, including subtypes H5N1 and H3N2, have been isolated from sparrows (*11*,*12*). A serologic survey also suggested that rates of influenza A virus infection were high among sparrows (*13*), which might result from abundant distribution of avian influenza virus receptor SA α2,3Gal in the respiratory tracts of sparrows (*14*). The novel subtype H7N9 virus expands not only the number of influenza virus subtypes that infect tree sparrows but also range of hosts for subtype H7N9 viruses. Our finding of only 1 subtype H7N9–positive sample among 2,198 samples is consistent with recent findings that subtype H7N9 in wild birds is rare (*15*).

Tree sparrows are abundant and widely distributed in China. They are frequently in contact with humans and poultry. Prevalence of avian influenza viruses among tree sparrows could increase opportunities for them to carry influenza viruses from aquatic birds to domestic farms and even to humans. Hence, such expansion of influenza A(H7N9) virus host ranges undoubtedly increased the seriousness of the threat of this novel subtype.

Tree sparrows have been shown to be susceptible to influenza A(H5N1) viruses, and they might have the ability to disseminate subtype H5N1 viruses (*11*). Dongping National Forest Park, where the novel subtype H7N9–positive tree sparrow was captured, is on Chongming Island, China's third largest island, which is located in the Australia–East Asia migratory wild bird flyway. Dongping National Forest Park is adjacent to Dongtan National Nature Reserve, where hundreds of species of migratory and domestic birds gather for winter. Whether migratory birds became infected through contact with tree sparrows and then disseminated subtype H7N9 virus to other geographic regions merits further investigation. Isolation of novel influenza A(H7N9) virus in a tree sparrow emphasizes the need to expand influenza surveillance to not only domestic birds but also wild and terrestrial birds.

Technical Appendix Sample sources (tissue types and geographic locations) and primer sets used for whole-genome PCR of influenza A(H7N9) virus.
