# Supplementary material for: Novel Avian Influenza A(H7N9) Virus in Tree Sparrow, Shanghai, China, 2013
Source: Emerg Infect Dis. 2014 May;20(5):850–3. doi: 10.3201/eid2005.131707 (PMC4012817; doi:10.3201/eid2005.131707)
Supplement: Technical Appendix — Sample sources (tissue types and geographic locations) and primer sets used for whole-genome PCR of influenza A(H7N9) virus. [file 13-1707-Techapp-s1.pdf]

Article DOI: http://dx.doi.org/10.3201/eid2005.131707

Novel Avian Influenza A(H7N9) Virus in Tree Sparrow, Shanghai, China, 2013

Technical Appendix

Technical Appendix Table 1. The information of sampling places in Shanghai, 2013

Sources of samples	wild migrate bird				wild domestic bird				bird in zoo		environment	unknown
	ardeidae	wader bird	W`ild geese and ducks	aquatic bird	wild chicken	sparrow	predator	owl	parrot	peacock	bird living environment	unknown
Swab	123	216	0	56	2	778	36	4	10	0	0	16
Feces	387	0	45	0	0	38	0	0	9	5	58	3
Tissues	0	0	0	0	2	333	65	4	0	0	0	8
Total	510	216	45	56	4	1149	101	8	19	5	58	27

Technical Appendix Table 2. Geographic distributions of sampling places in this study

	wild migrate bird				wild domestic bird				bird in zoo		environment	unknown
Sampling places	ardeidae	wader bird	wild geese and ducks	aquatic bird	wild chicken	sparrow	predator	owl	parrot	peacock	bird living environment	unknown
Baoshan district (BS)	50	0	45	0	0	2	1	0	0	5	0	0
Chongming district (CM)	55	216	0	48	0	313	10	0	0	0	34	0
Fengxian district (FX)	0	0	0	0	0	20	2	0	0	0	0	2
Jiading district (JD)	199	0	0	0	0	0	0	0	0	0	1	0
Jinshan district (JS)	52	0	0	0	0	0	2	0	0	0	0	0
Minhang district (MH)	50	0	0	0	0	0	0	0	0	0	0	0
Pudong district (PD)	28	0	0	6	4	709	82	8	0	0	0	20
Qingpu district (QP)	26	0	0	0	0	34	2	0	0	0	11	2
Shanghai center (SH center)	50	0	0	2	0	71	2	0	19	0	12	3
Total	510	216	45	56	4	1149	101	8	19	5	58	27

Technical Appendix Table 3. The primer sets for H7N9 whole genome PCR

Target gene	1st round RT-PCR Primers	2nd round PCR Primers	Forward primer name	Sequence	Reverse Primer name	Sequence
HA	1F/1680R	1F/693R	HA1F	ATGAACACTCAAATCCTGGTATTCG	HA7-1680R	TTATATACAAATAGTGCACCGCATGTTTC
		589F/1157R	HA7-589F	ACTGCAGAGCAAACCAAGCTATATG	HA7-693R	TTGTGGTCTCGCTCCTGGACT
		1043F/1218R	HA7-1043F	TCATTGAAAATGGATGGGAAGG	HA7-1218R	GTCTATCAACTCAAATTGTTGGTTGGT
		1043F/1680R	HA7-1680R	TTATATACAAATAGTGCACCGCATGTTTC	HA7-1157R	TGATCAATTGCCGATTGAGTGC
NA	NA1F/1398 R	1F/827R	NA9-1F	ATGAATCCAAATCAGAAGATTCTATGC	NA9-827R	GAGCATTCTTCAATATGCTTAGCAG
		790F/1271R	NA9-790F	TCTCTGACTGGAACTGCTAAG	NA9-1271R	ATCAACTCCACATAAAAACACG
		790F/1398R	NA9-1398R	TTAGAGGAAGTACTCTATTTTAGCCCC		
PA	PA1F/G215 1R	1F/708R	PA-1F	ATGGAAGAYTTTGTGCGAC	PA-708R	GAATCCATCCACATAGGCTCT
		13F/896R	PA-13F	GTGCGACAGTGCTTCAATCCA	PA-896R	CTTCGTGGCTCGGGTCCTCA
		688F/1550R	PA688F	AGAGCCTATGTGGATGGATTC	PA-1550R	ACGTCGGTATCATTCCTCAA
		1531F/2017R	PA1531F	TTGAGGAATGATACCGACGT	PA-2017R	GCTACTCATTGTTCAGGCGCTTA
		G1465F/G2151R	PAG1465F	TGTAGAACCAAAGAAGGAAGACG	PAG2151R	CTATCTTAGTGCATGTGTGAGGAAGG
PB1	PB1F/2274 R	1F/873R	PB1-1F	ATGGATGTCAATCCGACTTT	PB1-873R	AGTCATCATCTTCCTCACAACA
		8F/851R	PB1-8F	TCAATCCGACTTTACTTTTCT	PB1-851R	TTTGCCAATTTAGCTTTCTTCTCA
		852F/1774R	PB1-852F	TGTTGTGAGGAAGATGATGACT	PB1-1774R	AAACCAACAGTCCTGCCTTC
		1755F/2274R	PB1-1755F	GAAGGCAGGACTGTTGGTTT	PB1-2274R	CTATTTTTGCCGTCTGAGCT
PB2	PB2F/2280 R	1F/833R	PB2-1F	ATGGAAAGAATAAAAGAACTAAAGAG	PB2-833R	GCCAACGGGTCTGCTGATACTGT
		802F/1390R	PB2-802F	AGAAGAGCAACAGTATCAGCAGA	PB2-1390R	ATATTCCGATCATCCCCATTACA
		1281F/1796R	PB2-1281F	GCTAAATCCCATGCATCAACTCCT	PB2-1796R	AGAACCCTCACGAACCCACTA
		1686F/2221R	PB2-1686F	TCAATGGTCCCAAGATCCTAC	PB2-2221R	AGTCCCGTTTCCGTTTCATCACCA
		G1968F/G2208R	PB2G1968F	CAACTACAACAAGGCAACCAAGAGG	PB2GG2208R	TTAATTGATGGCCATCCGAATCC
			PB22280R	ATTCGACACTAATTGATGGC		
NP	1F/G1497R	1F/642R	NP-1F	ATGGCGTCTCAAGGCACCAAACGATC	NP-624R	CCTTCTTCCATTTTCGCCTCTCCAGA

Target gene	1st round RT-PCR Primers	2nd round PCR Primers	Forward primer name	Sequence	Reverse Primer name	Sequence
		485F/1081R	NP-485F	GGATGTGCTCTCTGATGCAAGGAT	NP-1081R	TAGTGGATAGCTGTCCTCTTGGGA
		926F/1292R	NP-926F	ACAGCCAGGTCTTTAGTCTCATTA	NP-1292R	CCTGTAAATGCTGCCATAATGGTTGC
		G1221F/G1497R	NPG-1221F	CGTTCAACCCACTTTCTCAGTA	NP-G1497R	TCAATTGTCATACTCCTCTGCATTG
NS	1F/838R	1F/471R	NSF1	ATGGATTCCAATACTGTGTCAAGCT	NS471R	CTACAATTGCTCCTTCTTCCGTAA
		1F/477R	NS447R	AGCTCTAAGTAGTATCAGGGCTT	NS381F	CATCACATTGAAAGCAAATTTC
		381F/838R	NS838R	TCATTAAATAAGCTGAAACGAG	NS342F	CCTATGCATTAGAATGGACCAAGC
		342F/791R	NS791R	CTTATCTCTTGCTCCACTTCAAGC		
M	F0/982R	1F/772R	MF0	AGCAAAAGCAGGTAGATG	M982R	TTACTTCAGCTCTATGTTGACA
		1F/521R	M1F	ATGAGTCTTCTAACCGAGGTCGAA	M772R	AACGACTAGAGGCTCACTTGAAC
		429F/982R	M521R	GCTAGTACCATTCTATTCTCATGC	M429F	TCTTGGACTAGTATGTGCCACTT
			M982R	TTACTTCAGCTCTATGTTGACA		

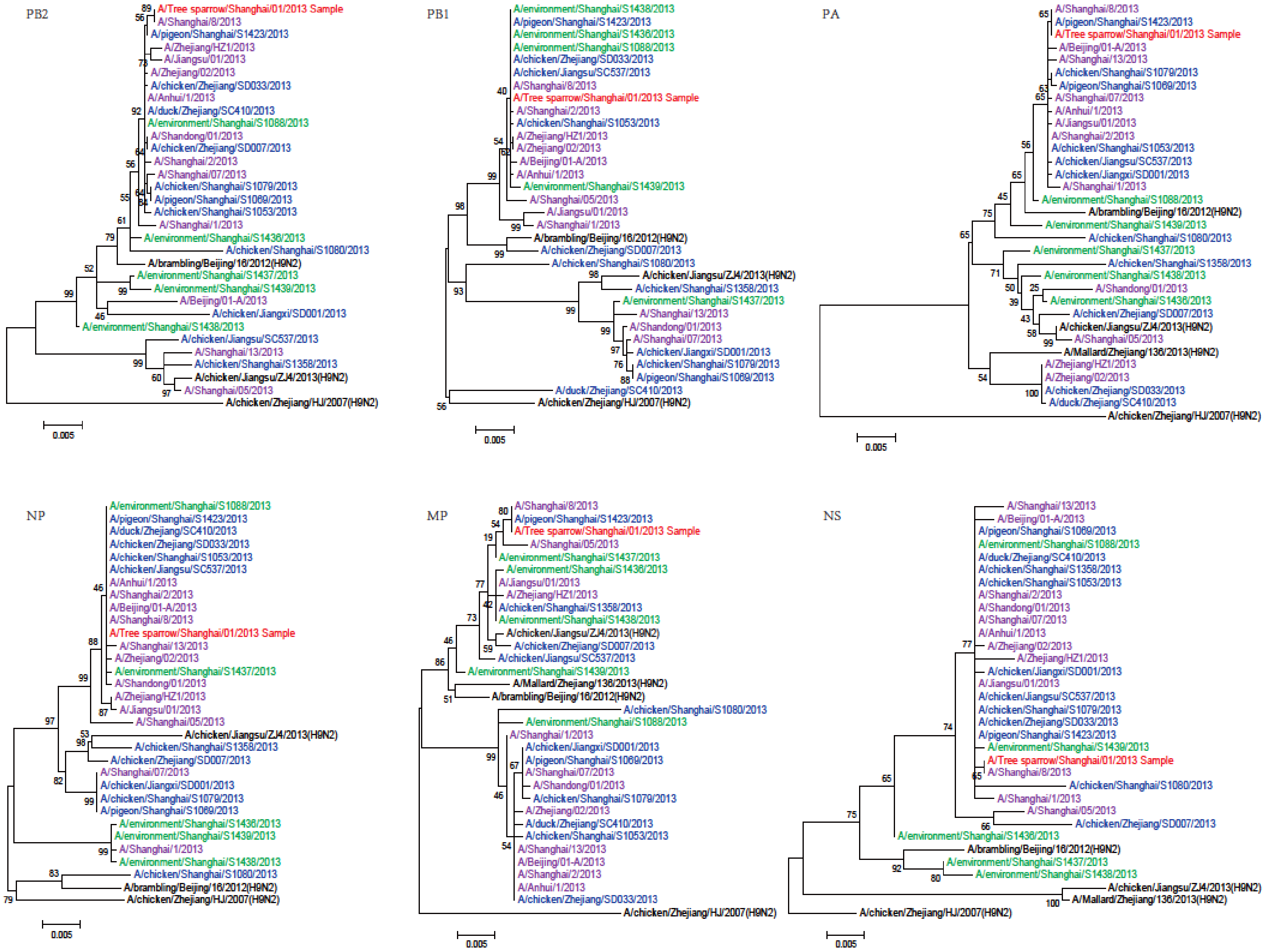
PB2
A/Tree sparrow/Shanghai/01/2013 Sample
A/Shanghai/8/2013
A/pigeon/Shanghai/S1423/2013
A/Zhejiang/HZ1/2013
A/Jiangsu/01/2013
A/Zhejiang/02/2013
A/chicken/Zhejiang/SD033/2013
A/Anhui/1/2013
A/duck/Zhejiang/SC410/2013
A/environment/Shanghai/S1088/2013
A/Shandong/01/2013
A/chicken/Zhejiang/SD007/2013
A/Shanghai/2/2013
A/Shanghai/07/2013
A/chicken/Shanghai/S1079/2013
A/pigeon/Shanghai/S1069/2013
A/chicken/Shanghai/S1053/2013
A/Shanghai/1/2013
A/environment/Shanghai/S1436/2013
A/chicken/Shanghai/S1080/2013
A/brambling/Beijing/16/2012(H9N2)
A/environment/Shanghai/S1437/2013
A/environment/Shanghai/S1439/2013
A/Beijing/01-A/2013
A/chicken/Jiangxi/SD001/2013
A/environment/Shanghai/S1438/2013
A/chicken/Jiangsu/SC537/2013
A/Shanghai/13/2013
A/chicken/Shanghai/S1358/2013
A/chicken/Jiangsu/ZJ4/2013(H9N2)
A/Shanghai/05/2013
A/chicken/Zhejiang/HJ/2007(H9N2)
0.005
PB1
A/environment/Shanghai/S1438/2013
A/pigeon/Shanghai/S1423/2013
A/environment/Shanghai/S1436/2013
A/environment/Shanghai/S1088/2013
A/chicken/Zhejiang/SD033/2013
A/chicken/Jiangsu/SC537/2013
A/Shanghai/8/2013
A/Tree sparrow/Shanghai/01/2013 Sample
A/Shanghai/2/2013
A/chicken/Shanghai/S1053/2013
A/Zhejiang/HZ1/2013
A/Zhejiang/02/2013
A/Beijing/01-A/2013
A/Anhui/1/2013
A/environment/Shanghai/S1439/2013
A/Shanghai/05/2013
A/Jiangsu/01/2013
A/Shanghai/1/2013
A/brambling/Beijing/16/2012(H9N2)
A/chicken/Zhejiang/SD007/2013
A/chicken/Shanghai/S1080/2013
A/chicken/Jiangsu/ZJ4/2013(H9N2)
A/chicken/Shanghai/S1358/2013
A/environment/Shanghai/S1437/2013
A/Shanghai/13/2013
A/Shandong/01/2013
A/Shanghai/07/2013
A/chicken/Jiangxi/SD001/2013
A/chicken/Shanghai/S1079/2013
A/pigeon/Shanghai/S1069/2013
A/duck/Zhejiang/SC410/2013
A/chicken/Zhejiang/HJ/2007(H9N2)
0.005
PA
A/Shanghai/8/2013
A/pigeon/Shanghai/S1423/2013
A/Tree sparrow/Shanghai/01/2013 Sample
A/Beijing/01-A/2013
A/Shanghai/13/2013
A/chicken/Shanghai/S1079/2013
A/pigeon/Shanghai/S1069/2013
A/Shanghai/07/2013
A/Anhui/1/2013
A/Jiangsu/01/2013
A/Shanghai/2/2013
A/chicken/Shanghai/S1053/2013
A/chicken/Jiangsu/SC537/2013
A/chicken/Jiangxi/SD001/2013
A/Shanghai/1/2013
A/environment/Shanghai/S1088/2013
A/brambling/Beijing/16/2012(H9N2)
A/environment/Shanghai/S1439/2013
A/chicken/Shanghai/S1080/2013
A/environment/Shanghai/S1437/2013
A/chicken/Shanghai/S1358/2013
A/environment/Shanghai/S1438/2013
A/Shandong/01/2013
A/environment/Shanghai/S1436/2013
A/chicken/Zhejiang/SD007/2013
A/chicken/Jiangsu/ZJ4/2013(H9N2)
A/Shanghai/05/2013
A/Mallard/Zhejiang/136/2013(H9N2)
A/Zhejiang/HZ1/2013
A/Zhejiang/02/2013
A/chicken/Zhejiang/SD033/2013
A/duck/Zhejiang/SC410/2013
A/chicken/Zhejiang/HJ/2007(H9N2)
0.005
NP
A/environment/Shanghai/S1088/2013
A/pigeon/Shanghai/S1423/2013
A/duck/Zhejiang/SC410/2013
A/chicken/Zhejiang/SD033/2013
A/chicken/Shanghai/S1053/2013
A/chicken/Jiangsu/SC537/2013
A/Anhui/1/2013
A/Shanghai/2/2013
A/Beijing/01-A/2013
A/Shanghai/8/2013
A/Tree sparrow/Shanghai/01/2013 Sample
A/Shanghai/13/2013
A/Zhejiang/02/2013
A/environment/Shanghai/S1437/2013
A/Shandong/01/2013
A/Zhejiang/HZ1/2013
A/Jiangsu/01/2013
A/Shanghai/05/2013
A/chicken/Jiangsu/ZJ4/2013(H9N2)
A/chicken/Shanghai/S1358/2013
A/chicken/Zhejiang/SD007/2013
A/Shanghai/07/2013
A/chicken/Jiangxi/SD001/2013
A/chicken/Shanghai/S1079/2013
A/pigeon/Shanghai/S1069/2013
A/environment/Shanghai/S1436/2013
A/environment/Shanghai/S1439/2013
A/Shanghai/1/2013
A/environment/Shanghai/S1438/2013
A/chicken/Shanghai/S1080/2013
A/brambling/Beijing/16/2012(H9N2)
A/chicken/Zhejiang/HJ/2007(H9N2)
0.005
MP
A/Shanghai/8/2013
A/pigeon/Shanghai/S1423/2013
A/Tree sparrow/Shanghai/01/2013 Sample
A/Shanghai/05/2013
A/environment/Shanghai/S1437/2013
A/environment/Shanghai/S1436/2013
A/Jiangsu/01/2013
A/Zhejiang/HZ1/2013
A/chicken/Shanghai/S1358/2013
A/environment/Shanghai/S1438/2013
A/chicken/Jiangsu/ZJ4/2013(H9N2)
A/chicken/Zhejiang/SD007/2013
A/chicken/Jiangsu/SC537/2013
A/environment/Shanghai/S1439/2013
A/Mallard/Zhejiang/136/2013(H9N2)
A/brambling/Beijing/16/2012(H9N2)
A/chicken/Shanghai/S1080/2013
A/environment/Shanghai/S1088/2013
A/Shanghai/1/2013
A/chicken/Jiangxi/SD001/2013
A/pigeon/Shanghai/S1069/2013
A/Shanghai/07/2013
A/Shandong/01/2013
A/chicken/Shanghai/S1079/2013
A/Zhejiang/02/2013
A/duck/Zhejiang/SC410/2013
A/chicken/Shanghai/S1053/2013
A/Shanghai/13/2013
A/Beijing/01-A/2013
A/Shanghai/2/2013
A/Anhui/1/2013
A/chicken/Zhejiang/SD033/2013
A/chicken/Zhejiang/HJ/2007(H9N2)
0.005
NS
A/Shanghai/13/2013
A/Beijing/01-A/2013
A/pigeon/Shanghai/S1069/2013
A/environment/Shanghai/S1088/2013
A/duck/Zhejiang/SC410/2013
A/chicken/Shanghai/S1358/2013
A/chicken/Shanghai/S1053/2013
A/Shanghai/2/2013
A/Shandong/01/2013
A/Shanghai/07/2013
A/Anhui/1/2013
A/Zhejiang/02/2013
A/Zhejiang/HZ1/2013
A/chicken/Jiangxi/SD001/2013
A/Jiangsu/01/2013
A/chicken/Jiangsu/SC537/2013
A/chicken/Shanghai/S1079/2013
A/chicken/Zhejiang/SD033/2013
A/pigeon/Shanghai/S1423/2013
A/environment/Shanghai/S1439/2013
A/Tree sparrow/Shanghai/01/2013 Sample
A/Shanghai/8/2013
A/chicken/Shanghai/S1080/2013
A/Shanghai/1/2013
A/Shanghai/05/2013
A/chicken/Zhejiang/SD007/2013
A/environment/Shanghai/S1436/2013
A/brambling/Beijing/16/2012(H9N2)
A/environment/Shanghai/S1437/2013
A/environment/Shanghai/S1438/2013
A/chicken/Jiangsu/ZJ4/2013(H9N2)
A/Mallard/Zhejiang/136/2013(H9N2)
A/chicken/Zhejiang/HJ/2007(H9N2)
0.005

Technical Appendix Figure 1. Phylogenetic trees of full-length polymerase basic (PB)2, PB1, polymerase acidic, nucleoprotein, matrix, and nonstructural genes of the tree sparrow–derived influenza A (H7N9) viruses in China, 2013. Sequences of human influenza A(H7N9) viruses are shown in purple, novel subtype H7N9 viruses from poultry (chickens, ducks, and pigeons) in blue, novel subtype H7N9 viruses from the environment in green, and novel subtype H7N9 viruses from wild birds in red. Scale bars indicate base substitutions per site.

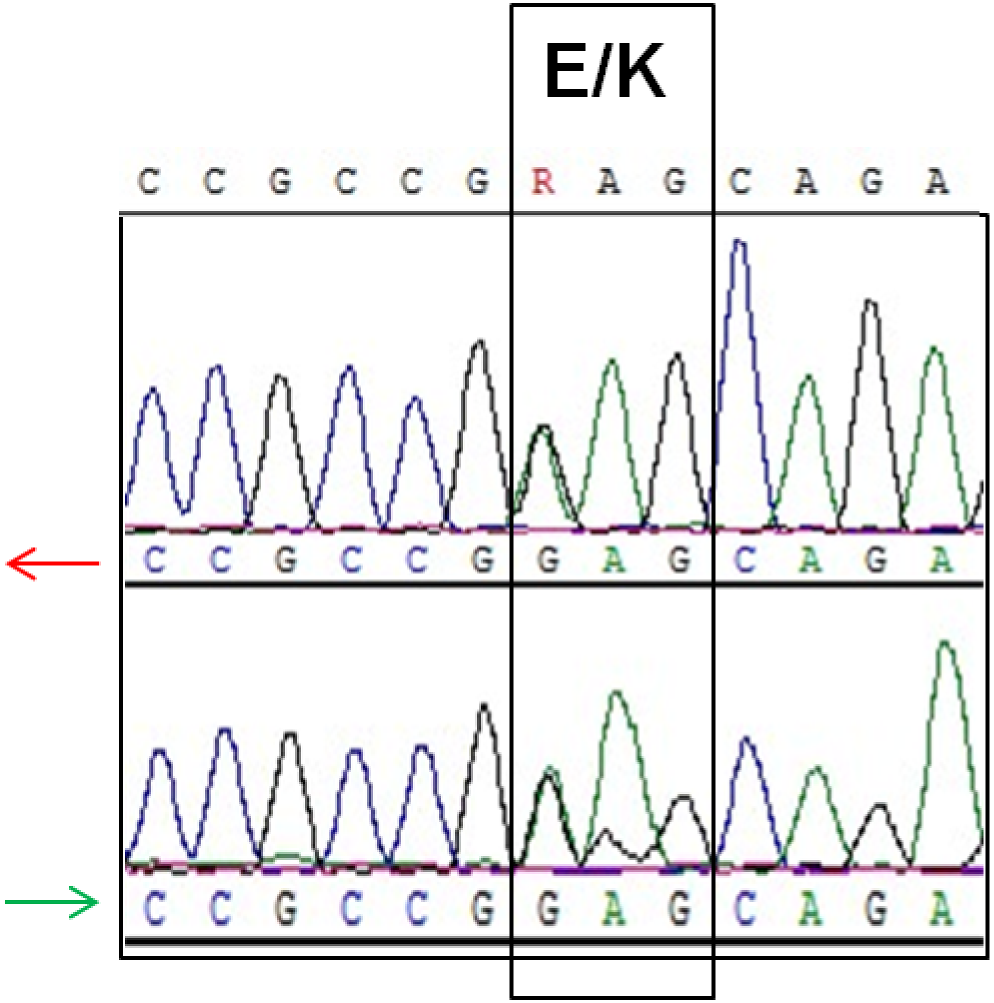

Technical Appendix Figure 2. Sequencing maps showing mixed population of 627E/K in polymerase basic 2 proteins from influenza A (H7N9) viruses. A, adenine phosphate (green); C, cytosine (blue); G, guanine (black). The square shows mixed nucleotides AAG (amino acid lysine, K) and GAG (amino acid glutamic acid, E). Red and green arrows indicated bidirectional sequencing.
